# Cell cycle and apoptosis in PER.C6^® ^cultures

**DOI:** 10.1186/1753-6561-7-S6-P48

**Published:** 2013-12-04

**Authors:** Sarah M Mercier, Bas Diepenbroek, Dirk E Martens, Rene H Wijffels, Mathieu Streefland

**Affiliations:** 1 Crucell, Leiden, The Netherlands; 2 Bioprocess Engineering, Wageningen University, Wageningen, The Netherlands

## Background

PER.C6^® ^is a human cell line designed for virus production, which was immortalized by transformation with adenoviral E1A and E1B genes. Expression of E1A is known to inhibit negative regulators of cell cycle and E1B protein function analogously to an apoptosis inhibitor. As changes in cell cycle and apoptosis are likely to affect cell's ability for viral infection and propagation, the study of these parameters in PER.C6^® ^cultures is essential to develop optimum virus production processes.

## Materials and methods

Cell cycle distribution and apoptosis were measured in batch and perfusion PER.C6^® ^cultures using flow cytometry. Propidium iodide was used to measure cell cycle distribution. Three methods were used to measure apoptosis: staining of externalized phosphatidylserine (PS) using annexinV, staining of activated caspases using a fluorochrome-conjugated inhibitor of caspases, and staining of fragmented DNA using BrdU incorporation and specific fluorescent labeling. 7-ADD was used to stain dead cells with a permeable membrane.

## Results

Significant cell death occurred in 14-days batches, when the main carbon sources were depleted. Apoptosis was initially not detected by the annexinV staining. However, activated caspases were detected after 6 days, suggesting that apoptosis occurred in batch. In perfusion, where the required nutrients were constantly supplied, no significant cell death or induction of apoptosis occurred, showing that the cultures were maintained in healthy conditions. At the end of batches, the portion of cells in S phase increased drastically and the one in G0/G1 decreased. In perfusion, cell cycle distribution was stable until 10 days, when a similar trend as the end of batch was observed.

This is the first research describing apoptosis and cell cycle distribution in PER.C6^® ^batch and perfusion cultures. Our data are in accordance with the theoretical effect of immortalization by the E1A/B system, which inhibits apoptosis when nutrients are in excess and promotes the entry into the cell division cycle.

